# Different pioneer plant species select specific rhizosphere bacterial communities in a high mountain environment

**DOI:** 10.1186/2193-1801-3-391

**Published:** 2014-07-29

**Authors:** Sonia Ciccazzo, Alfonso Esposito, Eleonora Rolli, Stefan Zerbe, Daniele Daffonchio, Lorenzo Brusetti

**Affiliations:** DeFENS, Department of Food, Environmental and Nutritional Sciences, University of Milan, via Celoria 2, 20133 Milan, Italy; Faculty of Science and Technology, Free University of Bozen-Bolzano, Piazza Università 5, 39100 Bolzano, Italy

**Keywords:** Rhizobacteria, Bacterial community structure, Pioneer plants, Glacier forefields, Alps

## Abstract

The rhizobacterial communities of 29 pioneer plants belonging to 12 species were investigated in an alpine ecosystem to assess if plants from different species could select for specific rhizobacterial communities. Rhizospheres and unvegetated soils were collected from a floristic pioneer stage plot at 2,400 m a.s.l. in the forefield of Weisskugel Glacier (Matsch Valley, South Tyrol, Italy), after 160 years of glacier retreat. To allow for a culture-independent perspective, total environmental DNA was extracted from both rhizosphere and bare soil samples and analyzed by Automated Ribosomal Intergenic Spacer Analysis (ARISA) and Denaturing Gradient Gel Electrophoresis (DGGE). ARISA fingerprinting showed that rhizobacterial genetic structure was extremely different from bare soil bacterial communities while rhizobacterial communities clustered strictly together according to the plant species. Sequencing of DGGE bands showed that rhizobacterial communities were mainly composed of Acidobacteria and Proteobacteria whereas bare soil was colonized by Acidobacteria and Clostridia. UniFrac significance calculated on DGGE results confirmed the rhizosphere effect exerted by the 12 species and showed different bacterial communities (*P* < 0.05) associated with all the plant species. These results pointed out that specific rhizobacterial communities were selected by pioneer plants of different species in a high mountain ecosystem characterized by oligotrophic and harsh environmental conditions, during an early primary succession.

## Background

In natural, agricultural, and forest ecosystems, rhizosphere is a biologically active zone of extreme importance because of the crucial role of plant–microorganism interactions in nutrient cycling, carbon sequestration, and ecosystem functioning (Singh et al. 2004; Hayat et al. 2010). The increasing interest in plant growth promotion, nutrition, and biological control of soil-borne plant pathogens requires a proper understanding of the structural and functional diversity of the bacterial communities in rhizosphere. Several studies have been carried out to reveal the impact of biotic and abiotic factors on the below-ground microbial diversity. It has been demonstrated that plant species and plant species composition select for taxonomic and functional groups in rhizosphere as a result of different root exudation and rhizodeposition. For instance, crops grown in monoculture or in agricultural soils have revealed evidence of plant species effect on specific bacterial communities (Duineveld et al. 1998; Grayston et al. 1998; Steer and Harris 2000; Smalla et al. 2001; Kowalchuk et al. 2002). Using a culture-independent analysis, specific bacterial communities have been observed in the rhizosphere of field-grown strawberry (*Fragaria ananassa* Duch.), oil-seed rape (*Brassica napus* L.), and potato (*Solanum tuberosum* L.). Moreover, plant species effect increased after planting the same crops in consecutive years (Smalla et al. 2001). However, studies of non-agricultural plant communities have indicated variable results. A high degree of plant effects was demonstrated for the native perennial bunchgrasses *Stipa*, *Hilaria* and for the invading annual grass *Bromus* (Kuske et al. 2002), for eight native herbaceous plants in Germany (Dohrmann and Tebbe 2005) or for *Nardo-Galion-* and *Lolio-Plantaginion* grasslands in Ireland (Brodie et al. 2003). Other experiments indicated the influence of both plant species and soil type (Marschner et al. 2001, 2004). Different studies showed that soil characteristics (Hansel et al. 2008; Wu et al. 2008; Kuramae et al. 2011, 2012), soil texture (Schutter et al. 2001), soil mineral composition (Carson et al. 2009), pH (Lauber et al. 2008), season, and land management (Kennedy et al. 2005) can exert a greater effect on rhizobacterial ecology than plant species and plant species composition.

In a natural ecosystem it is difficult to assess the effect of vegetation on rhizobacterial communities, especially in high mountain environments characterized by variable environmental parameters (i.e. successional stage, pH, rainfall, moisture, mineral composition, sampling season, slope) within a size-limited area typical of early and transitional successional stages. In a successional chronosequence resulting from a continuous glacier retreat patchy vegetation can colonize harsh environmental niches with high fraction of coarse-grained mineral skeleton, low total carbon and nitrogen content, and severe climatic regimes (Mattheus 1992). In glacier moraines, dynamic, severe environmental parameters could be expected to mask weaker rhizosphere effects in tiny pioneer plants, making an assessment difficult when linking plant species and rhizobacterial communities. The interaction between soil microbes and plants after glacier retreat might provide an approach to understand the biotic–abiotic interplays in primary succession, to develop strategies for sustainable protection of oligotrophic soils, and to identify the main factors in the formation of soils with high level of fertility (Doran, 2002). In early ecosystem development, plant colonization dramatically alters soil microbial community composition and function in many ways, and the plant-microbes interaction might increase the tolerance against strong abiotic constraints such as intense nutrient limitation. We can hypothesize that pioneer plants, which colonize early transitional successional stages, could select different rhizosphere microbial communities able to promote plant growth in these oligotrophic conditions. However only a few studies have examined how rhizosphere directly impacts microbial communities in young alpine ecosystems (Tscherko et al. 2004, 2005; Edwards et al. 2006; Miniaci et al. 2007). The major focus of these studies was to highlight the relationship between the chronosequences in alpine ecosystems and different microbial communities in the rhizosphere of pioneer plants and related bare soil. For instance, in an early successional stage, the rhizosphere microbial community of *Poa alpina* L. was strongly influenced by harsh abiotic constrains, but under more favorable environmental conditions, the plant could select for a more specific microbial community (Tscherko et al. 2004). Interestingly, along a similar chronosequence, the pioneer plant *Leucanthemopsis alpina* (L.) Heywood exerted a contradictory rhizosphere effect showing a specific microbial community only in the early succession stage (Edwards et al. 2006). However, the study of the spatial extent of *Lc. alpina* on the microbial community and physical-chemical parameters in an early successional stage (5, 10 years) did not exhibit significant trends, supporting the conclusion of Tscherko et al. (2004). Moreover, Tscherko et al. (2005) did not clearly show a selective effect of different plant species on the bacterial communities in the rhizosphere due to the effect that it was seemingly related to soil age.

The aim of this work was to assess if different plant species were able to select specific rhizobacterial communities in such a high mountain ecosystem. Consequently, we designed the experimental sampling plan to minimize, as much as we could, all the environmental variables (i.e. rainfall, moisture, sampling season) within a size-limited area characterized by same soil and ecological parameters (i.e. soil age, floristic successional stage, pH, moisture, mineral composition). We chose plants growing on 160 year old soil due to the quick glacier melting in the last 80 years, as it represents the only transitional step of our glacier moraine between earliest stages (<10 years) and mature soil (>500 years). As shown by aerial photos, ortophotos, and a topographic survey, the Matsch Glacier, one of the glacier tongues of the Weisskugel Glacier, has been retreating due to discontinuous movement. Therefore, we did not have a constant gradient of soil age, but rather distinct block stages in which soil age is invariable. Moreover, the 160 year old stage is characterized by harsh environmental niches with poor mineral skeleton, low total carbon, low nitrogen content, and severe climatic regimes. In this environment, more stable than earlier successional soil, it was possible to find a larger number of plant species individuals not interrelated in proto-floristic complex communities.

## Results

ARISA provided the fingerprints of both rhizosphere and bare soil bacterial communities. Due to the high sensitivity of the automated sequencer, complex profiles with peaks ranging from 155 bp to 1477 bp were obtained and the Internal Transcribed Spacer region (ITS) richness varied from 29 to 166 peaks.

**Figure 1:**
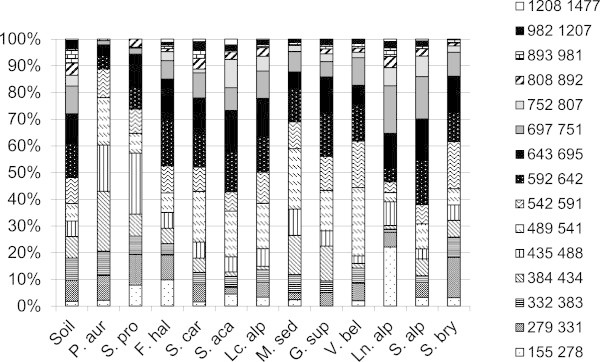
**ARISA peaks relative abundance within samples.** Stacked barcharts representing the relative abundance of group of ARISA peaks given in base pairs (bp), associated to the 12 pioneer plant species rhizosphere and to the unvegetated soil. Acronyms list name as follows: unvegetated soil; *Potentilla aurea*; *Sibbaldia procumbens*; *Festuca halleri*; *Senecio carniolicus*; *Silene acaulis*; *Leucanthemopsis alpina*; *Minuartia sedoides*; *Gnaphalium supinum*; *Veronica bellidioides*; *Linaria alpina*; *Sedum alpestre*; *Saxifraga bryoides.*

The electropherograms, characterized by distinct peaks number and intensity, revealed a large shift in bacterial community structure across the different plant species (Figure 1). The unvegetated soils revealed a high degree of similarity among replicates. They were different from rhizobacterial communities’ structure and more than 92% of their peaks were evenly distributed between 279–981 bp. The rhizobacterial communities showed distinctive peak patterns according to plant species. *Ln. alpina* exhibited a profile characterized by more than 20% of peaks between 155–280 bp and the most of bands distributed up to 643 bp. *F. halleri* had 10% of peaks between 155–280 bp, but more evenly distributed peaks until 807 bp, while *G. supinum* is the only species without peaks between 155–280 bp. Peaks between 279–331 represents 16% in *S. bryoides*. Peaks between 384–434 bp were the major peaks in *P. aurea* whereas peaks between 435–488 bp were the most abundant in *S. procumbens* and peaks between 489–541 bp were present in good percentage in *P. aurea*, *S. carniolicus*, *S. acaulis*, *Lc. alpina*, *V. bellidioides* and *M. sedoides*. Anyway, *S. acaulis* and *S. alpestre* were mainly characterized by peaks up to 592 bp. Comparing the unvegetated soil samples with all the 29 rhizosphere samples, PERMANOVA confirmed significantly different microbial community structures (F = 1.58; P < 0.007). Pairwise comparison of bulk soil and single plant species showed that pioneer plant species hosted a specific rhizobacterial community, except for *G. supinum* and *V. bellidioides* (P = 0.094; Table 1).

**Table 1:** **P values of the significance test from UniFrac web server (upper right side) and from PERMANOVA (lower left side) pairwise comparison of rhizosphere bacterial diversity between plant species**

Plant	***F. hal***	***G. sup***	***Lc. alp***	***Ln. alp***	***M. sed***	***P. aur***	***S. bry***	***S. alp***	***S. car***	***S. pro***	***S. aca***	***V. bel***	Soil
*F. hal*		0.30	0.60	0.14	0.44	0.50	0.50	0.29	0.29	0.02*	0.40	0.41	0.01*
*G. sup*	0.03*		0.85	0.37	0.05*	0.09	0.00*	0.04*	0.28	0.00*	0.44	0.30	0.02*
*Lc. alp*	0.01*	0.02*		0.62	0.60	0.44	0.06	0.51	0.91	0.01*	0.97	0.27	0.52
*Ln. alp*	0.02*	0.02*	0.01*		0.00*	0.02*	0.00*	0.02*	0.25	0.00*	0.33	0.01*	0.01*
*M. sed*	0.01*	0.01*	0.01*	0.02*		0.63	0.75	0.02*	0.00*	0.01*	0.01*	0.21	0.00*
*P. aur*	0.02*	0.02*	0.02*	0.02*	0.03*		0.14	0.23	0.28	0.21	0.22	0.47	0.10
*S. bry*	0.02*	0.01*	0.01*	0.01*	0.02*	0.02*		0.70	0.00*	0.58	0.23	0.13	0.00*
*S. alp*	0.01*	0.02*	0.01*	0.01*	0.01*	0.01*	0.01*		0.00*	0.00*	0.04*	0.02*	0.01*
*S. car*	0.02*	0.01*	0.02*	0.01*	0.01*	0.01*	0.01*	0.01*		0.00*	0.46	0.11	0.01*
*S. pro*	0.02*	0.02*	0.02*	0.02*	0.02*	0.01*	0.01*	0.02*	0.02*		0.00*	0.07	0.00*
*S. aca*	0.02*	0.03*	0.02*	0.02*	0.01*	0.02*	0.01*	0.01*	0.01*	0.02*		0.02*	0.02*
*V. bel*	0.01*	0.12	0.01*	0.01*	0.01*	0.02*	0.02*	0.01*	0.02*	0.02*	0.02*		0.00*
Soil	0.01*	0.01*	0.01*	0.01*	0.02*	0.01*	0.02*	0.02*	0.02*	0.01*	0.01*	0.02*	

P values were Bonferroni corrected. Full name of each plant species is reported into the text. Asterisks indicate significant values (P = 0.05).

**Figure 2:**
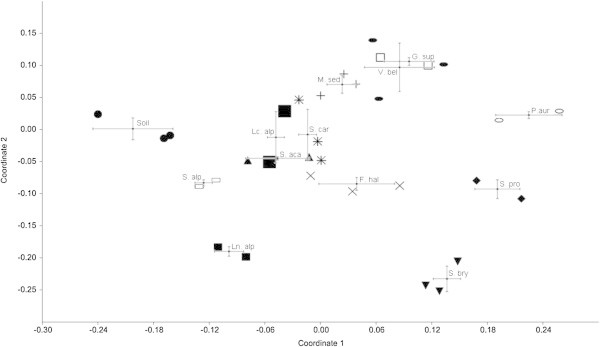
**Bacterial community structure by ARISA fingerprinting analysis.** Non-Metric Multidimensional Scaling analysis of the ARISA patterns of the bacterial communities associated to the 12 pioneer plant species rhizosphere and to the unvegetated soil. Symbols are as follows: black circle - bare soil; little black square - *Linaria alpina*; big black square - *Leucanthemopsis alpina*; black triangle - *Silene acaulis*; cross - *Festuca halleri*; asterisk - *Senecio carniolicus*; plus - *Minuartia sedoides*; white square - *Gnaphalium supinum*; black oval - *Veronica bellidioides*; white oval - *Potentilla aurea*; black diamond - *Sibbaldia procumbens*; white rectangle - *Sedum alpestre*; black wedge - *Saxifraga bryoides*. Values are means ± SD.

We studied the pioneer plants using a NMDS analysis based on Bray Curtis similarity measure (Figure 2). Stress value of NMDS analysis was 0.17. On the NMDS plot with the standard deviation, the replicated samples of bare soils are separated by the replicated samples of all the 12 plant species, indicating that each pioneer plant was able to modify the soil bacterial community and select specific rhizobacterial taxa. Five out of twelve plants, i.e. *G. supinum/V. bellidioides* and *Lc. alpina*/*S. carniolicus*/*S. acaulis*, grouped together, indicating a similar rhizobacterial community structure.

**Figure 3:**
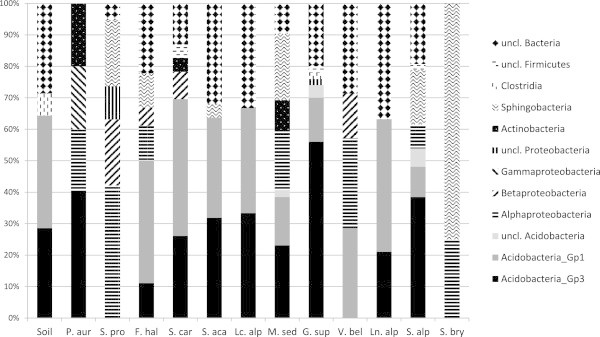
**Taxa identification by 16S rRNA gene DGGE-PCR.** Stacked barcharts representing the relative abundance of each bacterial taxon in bare soil and in the rhizosphere of the 12 pioneer plants. Plant names are as listed in Figure 1.

DGGE was performed to investigate the different microenvironments in terms of their dominant bacterial population. A total of 250 sequences of more than 300 bp were obtained, and RDP facilitated the determination of putative taxonomic affiliation of the recovered sequences. Major bacterial taxa included Acidobacteria Gp3 and Gp1, α-Proteobacteria, β-Proteobacteria, γ-Proteobacteria, Sphingobacteria, Actinobacteria, and Firmicutes (Figure 3). Members of Acidobacteria were present in all the rhizosphere and bare soil samples except in *S. bryoides* and *S. procumbens* rhizospheres. They were the most abundant taxa up to 74% of the 23 obtained sequences in *G. supinum*. We also found Proteobacteria in almost all the samples, except within *Ln. alpina*, *Lc. alpina* and *S. acaulis*, which was less abundant than Acidobacteria, except for *V. bellidioides*. However, Proteobacteria represented 74% of the 21 obtained sequences of the rhizosphere bacterial communities in *S. procumbens*. Sphingobacteria were 75% of the 19 obtained sequences of the bacterial communities associated to *S. bryoides* but they were recovered in a few of the samples with lower percentages. Members of Firmicutes and Actinobacteria taxa were even less abundant, being present in four and three plant species, respectively. We did not find Proteobacteria, Sphingobacteria and Actinobacteria associated to bare soil samples. Despite bias associated with sampling, DNA extraction, PCR amplification, and DGGE run, the pattern of differences in bacterial communities between bare soils and plant rhizosphere found by ARISA analysis were supported by the DGGE results.

**Figure 4:**
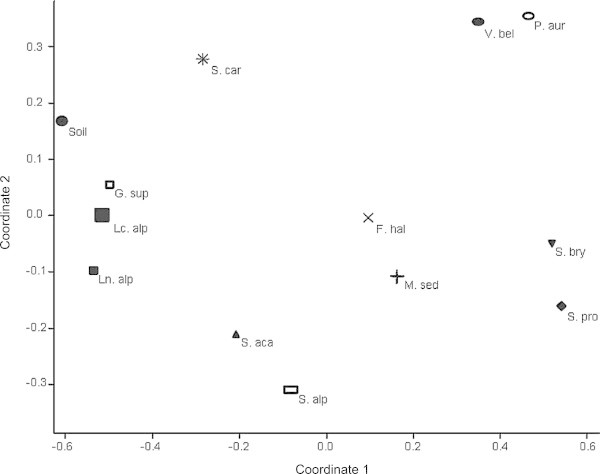
**Distribution of plants through DGGE bacterial diversity.** Non-Metric Multidimensional Scaling analysis of the 12 pioneer plants and the bare soil according to UniFrac distance matrix. Plant names are as listed in Figure 1.

Figure 4 shows the NMDS plot obtained by the weighted UniFrac distance matrix (stress value = 0.09). The wide distribution of the plant species in all the quadrants suggested different rhizobacterial community composition. Only *Ln. alpina, Lc. alpina* and *G. supinum* clustered more strictly together to the bare soil along the first axis, although *Ln. alpina* was separated from the unvegetated soil on the second axis.

Comparing each pair of environments using the significance tests of the UniFrac web server (*P* values < 0.05), bare soils samples were significantly different from almost all the rhizosphere (except for *Lc. alpina* and *P. aurea*; Table 1). Pairwise contrast of each single plant species showed that some pioneer plant species such as *S. procumbens* or *S. alpestre* were characterized by a more specific rhizobacterial community significantly different from most of the other plants, whereas other plant species such as *F. halleri*, *Lc. alpina*, and *P. aurea* showed a less divergent bacterial community composition.

Alpha and beta diversity analyses of both ARISA and DGGE results were calculated according to Whittaker (1960). Within each species, the averaged alpha diversity values were 0.64 ± 0.09 and 0.43 ± 0.33 for ARISA and DGGE results, respectively, whereas between species the averaged beta diversity value was 0.75 ± 0.07 for ARISA and 0.49 ± 0.21 for DGGE. Within bare soil samples, the averaged alpha diversity values was 0.73 ± 0.09 for the ARISA results, whereas it was 0.44 ± 0.00 for DGGE. Between soil and the twelve plant species, the averaged beta diversity values were 0.75 ± 0.04 and 0.46 ± 0.25 for ARISA and DGGE results, respectively.

## Discussion

This study supports the capability of pioneer plants from different species to select a specific rhizobacterial community in high-altitude alpine ecosystems. Our overall data of rhizobacterial community structure showed distinct clustering of bacterial communities according to plant species and well-differentiated with bare soil. These findings were confirmed by statistics on ARISA and DGGE by PERMANOVA and UniFrac downstream analysis, suggesting specialized genotypes adapted to each single rhizosphere conditions.

The harsh environmental conditions of high mountain environments can be very useful to highlight the role of the plants in the selection of specific rhizobacterial communities. The alternation of cold temperature during nights or in winter, followed by high temperatures at soil level in summer, as well as extreme levels of drought, rain, wind, snowfall, and irradiation result in only specialized plants’ survival and growth. Under a stable developmental stage condition, pioneer plants are evolutionarily adapted to these high mountain environmental conditions and hence could select their own bacterial communities under highly oligotrophic conditions. This is the case of our stable block stage of 160 years, which is the only transitional step of Matsch Valley glacier moraine.

Within the 16S-23S rRNA ITS, according to Fisher and Triplett (1999) and Ranjard et al. (2000), many Gram positive organisms have ITS lengths of about 400 bp, due to the absence of tRNA genes (Gurtler and Stanisich 1996), while ITS sizes of more than 500 bp belong to Gram negative bacteria. Our ARISA profiles suggested that some plant species like *S. bryoides*, *F. halleri*, and *M. sedoides* had an even distribution of Gram positive and Gram negative bacteria within the rhizosphere bacterial communities. On the other hand, *S. alpestre*, *Lc. alpina*, *V. bellidioides*, *S. acaulis*, *G. supinum,* and *S. carniolicus* hosted bacterial communities dominated by Gram negative bacteria, whereas *S. procumbens* and *P. aurea* showed an important presence of Gram positive bacteria. *Ln. alpina* showed a majority of ITS size below 280 bp, supporting Tscherko et al. (2005), who showed that Gram positive organisms are predominantly associated to *Ln. alpina* rhizosphere. Members of Acidobacteria and Proteobacteria phyla (Gram negative bacteria) were widely distributed in our samples, confirming the strict ecological and physiologic relationship to support ecological niches like rhizosphere soils (Smit et al. 2001; Quaiser et al. 2003).

The high incidence of Proteobacteria and Acidobacteria in sequencing of DGGE bands confirmed the importance of both phyla in mountain acid and oligotrophic soils, in opposition to plants of agricultural soils that showed predominance of Actinobacteria and Firmicutes (Smalla et al. 2001). The absence of major bacterial taxa in some rhizospheres and bare soil could be explained by the soil chemistry of the acidic ferrous rocks which characterize the valley. In a previous study about five different environmental matrices from Matsch Valley (Esposito et al., 2013), authors found similar patterns. For instance, Proteobacteria were rare in bare soils, representing less than 10% on average, while they were much more abundant in biofilms (40%) or in lichen thalli (50%). Also Acidobacteria showed a quite uncommon trend, with more presence in soils (40%) and no detection in biofilms. Other studies involving single pioneer plants showed the scattered microbial diversity maybe due to pioneer plants exudation and/or to mineral characteristics of the microsites (Edwards et al., 2006). The general role of pioneer plants in selecting their own bacterial communities from the overall bacterial taxa available in the surrounding environment is highlighted by our finding that of a decrease of alpha diversity values (of both ARISA and DGGE results) within replicated rhizospheres compared to beta diversities between plant species or plants and unvegetated soil.

Despite the different glacial forefield dynamics and the dissimilar early primary succession events, Knelman et al. (2012) demonstrated plant species’ effect on bacterial communities of early colonizer forest plants *Alnus sinuata, Picea sitchensis* in the Mendenhall Glacier forefield (AK, USA) after only six years of glacier retreat. Kuske et al. (2002) demonstrated an influence of plant species comparing the rhizosphere bacterial communities of three plant species of high mountain grasslands. To the best of our knowledge, these studies are the only studies available that demonstrate clear plant species’ effects in high mountains. Nevertheless, our results confirmed their conclusions about plant species’ selection in rhizobacterial communities, extending these dynamics to herbaceous isolated pioneer plants. Further studies, that investigated single plant species along alpine chronosequences (Damma Glacier, Switzerland) showed inconsistent results. For instance, Edwards et al. (2006) found that rhizosphere bacterial communities of the pioneer plant *Lc. alpina* were different from those of the interspace in an early successional soil, whereas in the late successional stage they were found to be more similar. Authors explained that the influence of *Lc. alpina* depended on soil age, linked to different nutrient availability. Additionally, Miniaci et al. (2007) studied some *Lc. alpina* individuals occurring after 5 and 10 years of glacier retreat. Miniaci et al. (2007) clearly demonstrate a rhizosphere effect, since some bacterial enzymatic activities and some soil chemical parameters were increased to 20 cm of distance to the plant, while bacterial biomass was evidently increased till 40 cm of distance. However they could not find any taxonomic shift between rhizobacterial communities through fingerprinting analyses of 16S rRNA gene (T-RFLP, DGGE) failing to demonstrate the selective effect of the *Lc. alpina* rhizosphere on its own microbial community. Another single pioneer plant studied along a chronosequence was *P. alpina* (Tscherko et al. 2004), which in the pioneer stage of soil formation it did not exhibit a selective role in its rhizosphere bacterial community. However, under more mature stages, this plant could select for a specific microbial community, significantly related to soil properties and carbon supply. Moreover, Tscherko et al. (2005) found that microbial biomass, rhizosphere enzyme activity, fungal/bacterial PFLA, Gram positives, Gram negatives and Gram positives/Gram negatives ratio were higher in late successional plant species than in early pioneer plants. But these findings did not conclusively prove a more pronounced plant species effect in late successional stage (75, 135 years) than in the first stage (43 years), since this result could also be explained by the effects of the different soil ages where plants grew.

One of the reasons for these different findings can be explained by the different technical approach. The determination of the PFLA profiles used by Tscherko et al. (2004, 2005) could only point out the different concentration of bacterial/fungal fatty acids and compare the Gram positives/Gram negatives ratio. Additionally, the DGGE analyses used by Edwards et al. (2006) and by Miniaci et al. (2007) normally gives less information on bacterial community structure than ARISA analysis. Whereas, in the present study, NMDS and PERMANOVA analyses of ARISA profiles and UniFrac significance values of sequencing of DGGE bands were found to be more successful to point out the differences in the ordination of samples structure among different plant species.

In our NMDS plot, the two clusters including *S. acaulis*/*Lc. alpina*/*S. carniolicus* and *G. supinum*/*V. bellidioides* can be explained because a successional chronosequence is not ecologically stable, being perturbed by occasional highly-disturbing events such as alluvial fans or screes, that take back the habitat to a pioneer stage, masking the weaker plant-selective role in shaping rhizosphere bacterial communities. In this case, random abiotic factors act as co-drivers of the rhizobacterial community development. Alternatively, plant species even taxonomically different could select for similar bacterial communities, at least on the basis of the overall genotype. This hypothesis is based on the knowledge that plant individuals can shape the rhizobacterial communities thanks to their root exudation (Smalla et al. 2001; Costa et al. 2006; Garbeva et al. 2008; Berg and Smalla 2009; Bonfante and Anca 2009). The enrichment of carbon of proto-soil by root exudates, as well as the release of hormones, are important shapers of the soil bacterial communities (Haichar et al. 2008; Huang et al. 2014). Since proto-soils immediately after glacier retreat are far from being sterile but host a particularly rich bacterial community (Steven et al. 2006; Rhodes et al. 2012; Edwards et al. 2013), the germinating seeds of the pioneer plants are in relation to a complex pool of soil bacteria adapted to live in mineral oligotrophic soils. Hence, through root exudation, plants select symbiotic, opportunistic or even pathogenic bacterial species, determining a spot of bacterial diversity near the root net (Berg and Smalla 2009; Ladygina and Hedlund 2010). Unfortunately to the best of our knowledge, the characterization of exudate profiles of wild pioneer plants is not well studied, and very little is known of the physiology of the plant species we found in the high Matsch Valley (Kytöviita et al. 2003; Ďugova et al. 2013; Roy et al. 2013). The increasing of bacteria by plants is a dynamic process that can be strengthened in the time after ice melting. Our soil has been under development since 1850, a quite long period that, in many other places can generate a complex soil with complex floristic consortia tending a more mature stage. But in the high Matsch Valley, the soil surrounding pioneer plant individuals is not really developed, delaying the transition to a more complex plant community. With such a very slow succession, plants and soil bacteria have enough time to co-develop influencing each other, and to increase the chance to obtain species-specific rhizobacterial communities.

## Conclusions

In conclusion, by using culture-independent methods, our research reveals that different plant species select for rhizobacterial communities different from those of the bare soil, despite the harsh environmental condition of the high-altitude alpine ecosystem. It will be interesting to investigate whether these pioneer plants, once associated in a proto-floristic community, are able to continue selecting specific bacterial communities in their rhizosphere according to plant species composition or whether this capability will be lost due to the interaction between counteracting root exudates.

## Methods

### Study site and soil samples

The study area is located in the upstream sub-catchment of the Saldur River (46° 46’ 30” N; 10° 41’ 46” E; 2,400 a.s.l.) in the high Matsch Valley (inner central Alps, South Tyrol, north Italy). The sub-catchment has a drainage area of 11 km^2^. The dominant geological processes are periglacial and the stream flow is dominated by the glacier dynamics.

The overall valley had an average precipitation of about 550 mm per year in the period of 1970–2000. In 2011, the mean temperature during the plant growing season was 7.3°C in July, 10.3°C in August, and 8°C in September and the mean precipitation was 2.7, 2.5, 3.6 mm per day, respectively. The main rock types are schist and gneiss (Habler et al. 2009), while the most diffused soil types are acidic leptosols, regosols, and umbrisols (mean pH = 4.3), derived from carbonate-free bedrocks. The study site is entirely located above the tree line (2,100 m a.s.l.), where a quick retreating glacier has left 3.3 km^2^ of foreland in the last 160 years (Knoll and Kerschner 2010). The different stages of glacier retreat were reconstructed by analyzing the historical maps of the Third Austro-Hungarian topographic survey (the so called “Franzisco-JosephinischeLandesaufnahme”) dated 1850, an aerial photograph of 1945, and ortophotos from 2006. On the basis of those photos, our sampling site has been ice-free since 1850.

Rhizosphere and soil sampling were carried out in September 2011, at the end of the plant growing season. One plot of 5 × 5 m was designed in order to be as homogeneous as possible in terms of altitude, aspect, slope, and physical and chemical composition. Soil was a sandy silt soil with humus, having an average texture of 72.3 ± 5.0% of sand, 21.0 ± 4.1% of silt, 6.6 ± 1.3% of clay, and 4.6 ± 1.3% of humus. Total organic carbon was 2.6 ± 0.8%, while pH was 4.5 ± 0.3%. The average chemical composition of the sampled soils was ammonia 3.4 ± 1.0 mg kg^−1^ d.m., total P 0.7 ± 0.1 mg kg^−1^ d.m., total K 7.4 ± 1.0 mg kg^−1^ d.m., total Ca 3.4 ± 0.6 mg kg^−1^ d.m., total K 7.4 ± 1.0 mg kg^−1^ d.m., total Mg 13.4 ± 1.7 mg kg^−1^ d.m., total Fe 45.4 ± 6.9 mg kg^−1^ d.m., and total Al 29.4 ± 5.6 mg kg^−1^ d.m. The measured values are expressed as average plus standard deviation. No nitrate and calcium carbonate were detected.

Twelve of the most representative plant species of the 160 year old successional stage were identified and sampled inside the plot: *Festuca halleri* All. (3 replicates), *Gnaphalium supinum* L. (2 replicates), *Leucanthemopsis alpina* (L.) Heywood (2 replicates), *Linaria alpina* (L.) Mill (2 replicates)*, Minuartia sedoides* (L.) Hiern (3 replicates)*, Potentilla aurea* L. (2 replicates)*, Saxifraga bryoides* L. (3 replicates), *Sedum alpestre* Vill. (2 replicates), *Senecio carniolicus* (Willd.) Braun-Blanq (3 replicates), *Sibbaldia procumbens* L. (2 replicates), *Silene acaulis* (L.) Jacq (2 replicates), and *Veronica bellidioides* L. (3 replicates) [Varolo E., personal communication].

We sampled at least two field replications of each plant species, avoiding the influence of the rhizosphere of other plant species and individuals. After pulling out each plant, we collected the soil adhering to the roots. An amount of about 4 g of rhizosphere soil for each plant was extracted. Three replicates of bare soil were collected from the same area as a control. Soil samples were put in sterile bags and transported in refrigerated boxes to the laboratory within 3 hours, and stored at −80°C until the analyses.

### Molecular analysis of the bacterial communities

Total DNA of the rhizosphere and soil samples was extracted using Ultraclean Soil DNA Extraction kit (MO-BIO, Arcore, Italy) following the manufacturer’s instruction. Microbial analyses were carried out using culturing-independent methods, i.e. DGGE (Muyzer et al. 1993) to describe the dominant bacterial taxa associated to each plant species and ARISA (Cardinale et al. 2004) to describe the structure of the overall rhizobacterial communities.

For the DGGE analysis, primers GC357f and 907r were used as described by Sass et al. (2001). The primer set 357f-907r is designed to be universal in the detection of Bacteria (Lane 1991; Muyzer et al. 1993). DGGE was run in a BioRad DCode Universal Mutation Detection System. Polyacrylamide gels were done according to Muyzer et al. (1993). The gels were stained for 30 min in 1 × TAE buffer containing SYBR® Safe - DNA Gel Stain (Invitrogen, Milan, Italy). Visualization and digital image recording was performed with UVTec (Cambridge, UK). DGGE bands were excised and reamplified as described (Muyzer et al. 1993). We excised and sequenced all the visible bands of the gels with an average of 18 bands per plant species.

Sequencing was performed by STAB-Vida Inc. (Caparica, Portugal). Identification of 16S rRNA genes was done by a comparison with the EMBL/Genebank/DDBJ database and RDP database using BLASTN and Classifier, respectively. All sequences were submitted to the RDP-Ribosomal Database Project (Cole et al. 2009) web server to assign taxonomy. Sequences were submitted to the Genbank/EMBL/DDBJ databanks under the accession numbers from HF930771 to HF931020.

ARISA fingerprint was performed as described elsewhere (Cardinale et al. 2004) with the ITSF/ITSREub primer set. The primer set was demonstrated to be universal, amplifying representatives of all the bacterial phyla (Cardinale et al. 2004) and no representatives of Archaea and Fungi. Denatured ARISA fragments were run by STAB-Vida Inc. The data were analyzed with Peak Scanner Software v1.0 (Applied Biosystems), and a threshold of 40 fluorescent units were used, corresponding to two times the highest peak detected during the negative control run. If the baseline varied inconsistently, the sample was rerun. Output matrix was obtained as by Rees et al. (2004).

### Statistical analysis

Multivariate analysis was performed on ARISA profiles that were normalized with the formula (x/∑x)*1000, where “x” is the fragment height in units of fluorescence and then log transformed to balance the advantage of analyzing non transformed data, which keeps relative abundance information, and binary data, which down-weigh abundant groups. In order to assess changes in rhizobacterial community structure between different plant species, nonmetric multidimensional scaling (NMDS) based on Bray Curtis similarity measure was applied to analyze unbalanced data of ARISA matrices with a high number of 0 and non-Gaussian distribution of peaks’ height/area. NMDS avoids the assumption of linear relationships among variables, and it is reported to be the most effective ordination method for ecological community data (Clarke 1993).

PERMANOVA based on Bray-Curtis similarity was performed to test significant differences in the profile composition between the plant rhizospheres and bare soils. PERMANOVA is a nonparametric permutation-based statistical test of significant differences between two or more groups, based on any distance measure (Anderson 2001). It is an analogue of the univariate ANOVA and it calculates the F value. The null hypothesis was rejected when the significance value was < 0.05. The values were corrected for multiple comparisons by multiplying them by the number of comparisons made (Bonferroni correction).

The UniFrac web server requires, as input, a single rooted phylogenetic tree which contains sequences from at least two different environmental samples. The Nexus format of the neighbor-joining phylogenetic tree, performed by MEGA5, using DGGE sequenced bands, was submitted to the UniFrac web server to test differences among each pair of environments based on the UniFrac metric (UniFrac significance) with 100 permutations (Hamady et al. 2010). The UniFrac metric calculates the difference between two environments in term of branch length that is unique to one environment or to another (Lozupone and Knight 2005). Moreover, we chose to use weighted UniFrac which weights the branches based on abundant information of different kinds of Bacteria. Environments are similar when the UniFrac value of the real tree is lower than would be expected if the sequences were randomly distributed between environments. The values were corrected by Bonferroni correction as above. The distance matrix for each pair of environments was obtained using the UniFrac metric and the nonmetric multidimensional scaling (NMDS) was performed using R environment (R Core Team, 2012).

All the statistics, including multivariate, alpha, and beta-diversity analyses, were performed using PAST program (Hammer et al. 2001). Ecological diversity indices were calculated on the normalized ARISA matrix and on the binary DGGE matrix.
